# Antigenic presentation of heterologous epitopes engineered into the outer surface-exposed helix 4 loop region of human papillomavirus L1 capsomeres

**DOI:** 10.1186/1743-422X-6-81

**Published:** 2009-06-18

**Authors:** Yoshihiko Murata, Paula M Lightfoote, Robert C Rose, Edward E Walsh

**Affiliations:** 1 Division of Infectious Diseases, Department of Medicine, University of Rochester School of Medicine and Dentistry, Rochester, New York 14642, USA; 2 Infectious Diseases Unit, Department of Medicine, Rochester General Hospital, Rochester, New York 14621, USA; 3 Department of Microbiology and Immunology, University of Rochester School of Medicine and Dentistry, Rochester, New York 14642, USA

## Abstract

**Background:**

Human papillomavirus (HPV) L1 capsid proteins can self-assemble into pentamers (capsomeres) that are immunogenic and can elicit neutralizing antibodies. Structural modelling of L1 inter-pentameric interactions predicts that helix 4 (h4) of each of the five L1 monomers project laterally and outwards from the pentamer. We sought to utilize HPV L1 capsomeres as a vaccine platform by engineering heterologous epitopes within L1 derivatives deleted for h4 domain.

**Results:**

We used baculovirus – infected *Trichoplusia ni *cells and ultracentrifugation to synthesize and purify three 16L1 derivatives: one bearing a short deletion (amino acids 404–436) encompassing the h4 domain, and two others, each bearing a conserved neutralizing epitope of the human respiratory syncytial virus (RSV) fusion (F) protein (residues 255–278 and 423–436) that was substituted for the deleted L1 h4 domain residues. Each of the three capsomere derivatives was recognized by anti-L1 antibodies, while two bearing the RSV F-derived moieties were recognized by anti-RSV F antibodies. All three L1 derivatives formed ring-like structures that were similar in morphology and size to those described for native 16L1 capsomeres. When injected into mice, each of the capsomere derivatives was immunogenic with respect to L1 protein, and immunization with chimeric L1-RSV F pentamers resulted in RSV non-neutralizing antisera that recognized purified RSV F protein in immunoblots.

**Conclusion:**

HPV L1 monomers bearing heterologous epitopes within the L1 h4 region can self-assemble into capsomeres that elicit antibody response against such non-HPV encoded epitopes. Thus, the L1 h4 region can function as a novel antigen display site within the L1 pentamer, which in turn may serve as a potential vaccine template.

## Background

Human papillomaviruses (HPVs) are non-enveloped DNA oncogenic viruses that cause significant burden of disease, including cervical dysplasia and cancer [1]. The major structural component of the HPV virion is the L1 viral capsid protein that can spontaneously form pentamers (capsomeres) [2,3]. Such L1 oligomers can then self-assemble into one of two virus-like particles (VLPs): a spherical lattice structure of T = 7 symmetry group that is morphologically indistinguishable from native HPV virions, or a smaller T = 1 particle that is comprised of 12 L1 pentamers and for which the crystal structure has been solved [3-5].

L1 VLP formation requires inter-capsomeric hydrophobic interactions involving helices 2, 3, and 4 (h2, h3, and h4, respectively) near the carboxy-terminus of each L1 monomer [3,5,6]. In L1 capsomeres, these helices project laterally and outwards onto the solvent-exposed surface. Helix 4 from a L1 monomer within a capsomere forms hydrophobic interactions with h2 and h3 of a L1 molecule of an adjacent capsomere to link the two L1 pentamers. Deletion of h4 has no obvious effect on L1 capsomere assembly, but abolishes the ability of L1 to form T = 1 or T = 7 VLPs [6].

In addition to its self-assembling capabilities, the papillomavirus L1 protein can function as potent immunogens when oligomerized as capsomeres and VLPs [4,7,8]. Bacterially derived L1 proteins from HPV type 16 (HPV-16) and other HPV serotypes as well as those derived from the oncogenic canine oral papillomavirus (COPV) form capsomeres in vitro and elicit neutralizing antibodies [9-12]. Immunization with COPV-L1 capsomeres generates a protective response in a subsequent COPV-canine oral mucosal challenge [10]. The L1 HPV VLPs elicit robust neutralizing and protective antibodies, and have recently been licensed as prophylactic vaccines against HPV infection [13,14].

The biophysical and immunological properties of HPV L1 capsomeres and VLPs suggest that these structures may function as vaccine platforms (reviewed in [15]). To this end, several studies have described the generation of chimeric VLPs bearing heterologous antigenic residues at the carboxy-terminus or surface-exposed loops of L1 monomers (e.g. [16-18]). However, the challenges of such approaches include inefficient antigen display, the limited structural capacity of L1 surface loops to accommodate foreign epitopes, and potentially significant disruption of L1 oligomeric structures. To circumvent these issues, we chose the L1 h4 domain as a novel antigen presentation site since this region is predicted to be surface-exposed in capsomeres. In place of the h4 and surrounding residues, we generated L1 derivatives bearing one of two previously characterized neutralizing epitopes of the RSV F protein [19]. We demonstrate that L1 derivatives bearing either of the two foreign epitopes can form oligomers that are morphologically similar to capsomeres. Furthermore, such modified L1 pentamers can elicit antibodies that recognize the RSV F protein.

## Results

### Expression and purification of HPV 16L1 derivatives bearing h4 deletion and substitutions

**Figure 1:**
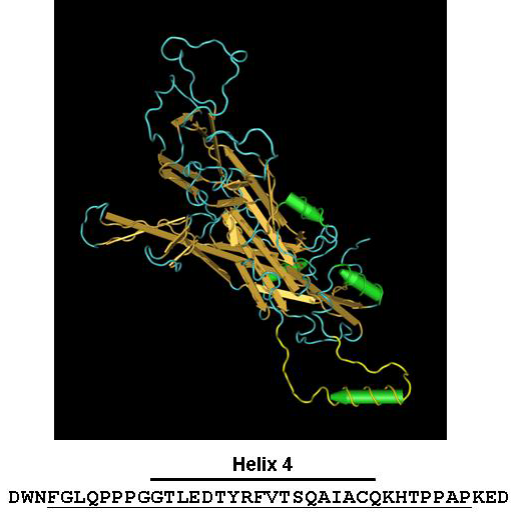
**The h4 domain of the HPV 16L1 monomer**. Shown is a ribbon diagram of the HPV 16L1 monomer that is visualized using the Cn3D program (NIH) and arranged such that the h4 domain is shown protruding out of the plane of the diagram. Shown below the schematic is the aa sequence of L1 h4 and its surrounding residues 401 – 439; those comprising h4 are highlighted by an upper bar over sequence, while aa 404–436 are underlined; the cognate portion within the ribbon diagram are highlighted in yellow.

**Figure 2:**
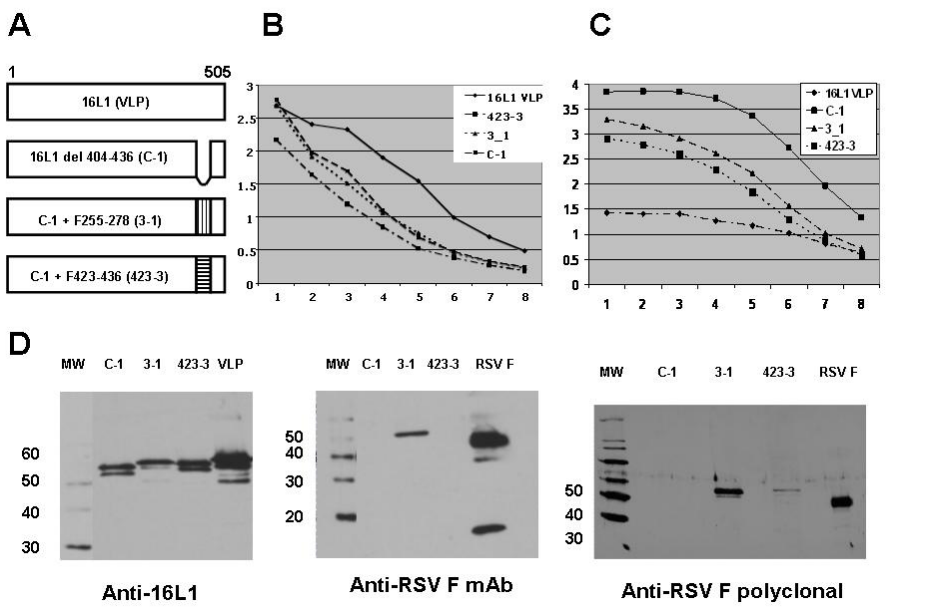
**Immunological and structural analyses of the L1 deletion and its derivatives**. A) Schematic diagram of the full-length 16 L1 protein (VLP) and three L1 derivatives: a deletion lacking residues 404–436 (C-1), and those bearing RSV F aa 255–278 (3-1) and 423–436 (423-3) within the C-1 deletion. B and C) ELISA analysis of L1 derivatives: mouse polyclonal anti-HPV 16 VLP (Panel B; starting at 1:10,000) and mouse mAb V5 (Panel C; starting at 1:100,000) were used to detect L1 VLPs and the capsomere derivatives. Horizontal axis represents serial two-fold dilutions of antibodies and vertical axis represents OD_405 nm _of the resulting reactions. D) Immunoblots of L1 VLP and capsomere derivatives. Except for the 423-3 lane in the right panel, ~0.5 μg/lane of L1 proteins were resolved on 10%/5% (left and right panels) or 12%/6% (middle panel) SDS-PAGE gels, transferred onto nitrocellulose, and detected with anti-16L1 mAb (CAMVIR-1;1:60,000 dilution), anti-RSV F neutralizing mAb (L4; 1:5,000 dilution), or anti-RSV F polyclonal rabbit serum (1:1,000 dilution) followed by either goat anti-mouse IgG-HRP or anti-rabbit IgG-HRP (at 1:20,000 dilution) and chemiluminescence. Molecular weight standards are shown to the left of each marker ladder. The two anti-RSV F antibodies used in this study recognize both the 50 kD and 20 kD subunits of F protein. Compared to other lanes, 3–4 fold more total protein of the 423-3 derivative was loaded; similar over-loading of C-1 preparations did not lead to increased non-specific recognition of capsomeres by the polyclonal anti-RSV serum (data not shown). The multiple L1 bands around 55 kD likely indicate minor differences in post-translational modifications of the L1 derivatives.

To identify h4-spanning portions of the L1 carboxy terminus region into which heterologous epitopes can be engineered, we first generated two deletions within L1: one that abolished all but the first residue of h4 (aa 413–430; termed B-1) and another that deleted h4 and additional surrounding residues, including the prolines flanking both sides of h4 (aa 404–436; C-1, Figure 1). HPV type 16 L1 protein and its cognate cDNA were used for all L1 derivatives in this study [20]. Into each of the L1 deletions, we placed two epitopes from the RSV A2 strain F protein: aa 255–278, which forms a helix-coil-helix structure in solution and is recognized by the neutralizing, fusion-inhibiting monoclonal antibody (mAb) L4 [21-23]; and aa 423–436, a linear epitope recognized by mAb 101 [24,25]. For each of the L1 deletions and its two RSV F epitope-bearing derivatives, baculoviruses programmed to express each protein was generated and used to infect *Trichoplusia ni *(*T. ni*) cells. Using isopycnic and sucrose cushion centrifugations, the resulting capsomere-enriched fractions were collected, dialyzed into high-salt buffer (PBS/1 M NaCl for long-term structural stability), and analyzed for protein yield and purity. We consistently observed that B-1 capsomeres and its two RSV epitope-bearing derivatives were of inferior quality and quantity and thus were not studied further (data not shown). In contrast, C-1 and its derivatives, 3-1 (bearing RSV F residues 255–278), and 423-3 (+ RSV F 423–436) were well expressed (schematically depicted in Figure 2A), enriched to > 80% purity (data not shown) and were used in subsequent experiments.

### Immunological and structural characterization of L1 derivatives

To determine whether the capsomere derivatives bore the expected L1 and RSV F-derived epitopes, we subjected the capsomere preparations to a series of immunological tests. In ELISAs, control L1 VLPs and all three capsomeres were recognized by a mouse polyclonal anti-L1 VLP antiserum as well as anti-HPV 16L1 mAb V5, which recognizes an immunodominant epitope within 16L1 (Figure 2B and 2C) [20,26]. Interestingly, as compared to L1 VLPs, all three capsomere derivatives were recognized more strongly by mAb V5. In immunoblots, the L1 derivatives were recognized by the CAMVIR-1 anti-HPV 16L1 mAb, with the C-1 deletion migrating slightly faster as anticipated and as compared to the L1 from VLPs or the 3-1 and 423-3 derivatives (Figure 2D). When tested for the presence of RSV F 255–278, the anti-RSV F mAb L4 recognized 3-1 but not C-1 or 423-3 in immunoblots (Figure 2D) or ELISAs (data not shown). The unavailability of an anti-RSV F mAb recognizing the second RSV F epitope (aa 423–436) within our antibody panel precluded similar analysis of 423-3. However, we were able to reproducibly detect the presence of 423-3 rabbit polyclonal anti-RSV F antisera (Figure 2D) [23]. Thus we conclude that all capsomere derivatives in this study exhibit no gross deficiencies in L1-derived epitopes, and that two L1 derivatives express the RSV F epitopes as expected. In addition, the ELISA data using V5 mAb suggest that the V5 binding site may be more readily accessible in capsomeres than in VLPs and that deletions and substitutions of L1 h4 may subtly affect accessibility and/or conformation of the V5 recognition site.

**Figure 3:**
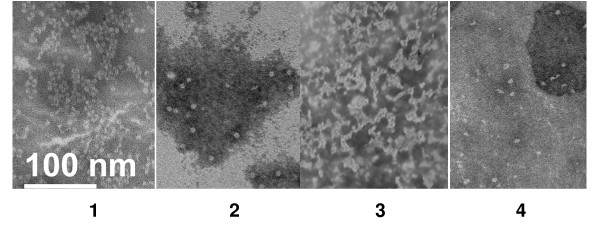
**Electron micrographs of L1 capsomere derivatives**. Purified proteins were placed on carbon-copper grids, treated with uranyl acetate, and visualized using transmission electron microscopy. The samples are: full-length L1 capsomeres generated from dithiothreitol treatment of purified L1 VLPs (Panel 1); C-1 (2); 3-1 (3); and 423-3 (4). The bar in Panel 1 represents the scale of the microscopy images.

**Figure 4:**
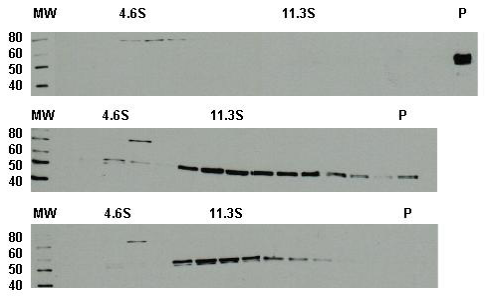
**Sucrose gradient analysis of L1 VLPs and capsomere derivatives**. Ultracentifugation with 5–20% sucrose gradient in PBS/1 M NaCl was used to resolve purified L1 VLPs (top panel), 3-1 (middle panel), and 423-3 (lower panel). For each fraction, 12 μl was resolved on 10%/5% SDS-PAGE, transferred onto nitrocellulose, and visualized using CAMVIR-1 anti-L1 mAb (1:60,000 dilution), goat anti-mouse IgG-HRP conjugate (1:20,000 dilution) and chemiluminescence. Molecular weights are shown to the left of the standard ladder. For each fractionation, sedimentation standards were concurrently resolved (bovine serum albumin, 4.6S; bovine catalase, 11.3S, and *E. coli *β-galactosidase, 19S). For the first two standards, the peak fractions for each gradient are shown on top of the panel; under our typical ultracentrifugation parameters (16–20 hours) and buffer composition with PBS/1 M NaCl as the sucrose solvent, we consistently observed that the 19S standard migrated to the bottom of the tube into the pellet (P) fraction. Note that the VLPs are found in the pellet fraction while the capsomere preparations were resolved across the gradient (see Results). Note also that there is an 80 kD band that is enriched around the 4.6S standard-containing fractions in all of our sucrose gradient-derived immunoblots (this Figure and data not shown). Since this band was not seen in immunoblots of pre-gradient L1 preparations, we assume that this represents an artifact from one or more of the sedimentation standards.

To examine the structure of capsomeres and to test its behavior in solution, we performed transmission electron microscopy (EM) and sucrose gradient analysis. All three capsomere preparations formed ring-like structures with diameters typically ranging from 7–10 nm, consistent with the morphology previously described for L1 capsomeres derived from VLPs (Figure 3) [27,28]. We occasionally noted that some capsomere preparations, especially those for 3-1, appeared to yield structures in variable states of aggregation (Figure 3, panel 3). This observation may have been due to either artifacts of EM sample preparation or our use of PBS/1 M NaCl in anticipation of subsequent mouse immunogenicity studies instead of buffers with lower pH (5–6) that favor capsomere formation [6]. Nonetheless, to ensure that the capsomere derivatives did not exist in primarily aggregated forms, we subjected our preparations to sucrose gradient ultracentrifugation. Using 5 – 20% sucrose gradients in PBS/1 M NaCl, we noted that the purified 16L1 VLPs pelleted at the bottom of the gradient, while the sedimentation peak for all three capsomeres was approximately 11S; these observations are in accord with previously reported fractionations of L1 VLPs and of native capsomeres under similar centrifugation conditions, respectively (Figure 4) [28]. We also observed limited amounts of capsomeres disassembled into L1 monomers (which would remain near the top of the gradient; Figure 4, bottom panel). Moreover, in gradient fractionations of 3-1 and C-1 (to a lesser degree), the capsomeres were localized in fractions with sedimentation coefficients > 11S (Figure 4, middle panel), presumably due to aggregation and consistent with EM analysis. Taken together, these biophysical characterizations suggest that the morphology and in-solution behavior of the three capsomere derivatives are similar to those of native L1 pentamers, although some aggregation appears to occur in a subset of capsomere preparations.

### Immunogenicity of HPV 16L1 derivatives

**Figure 5:**
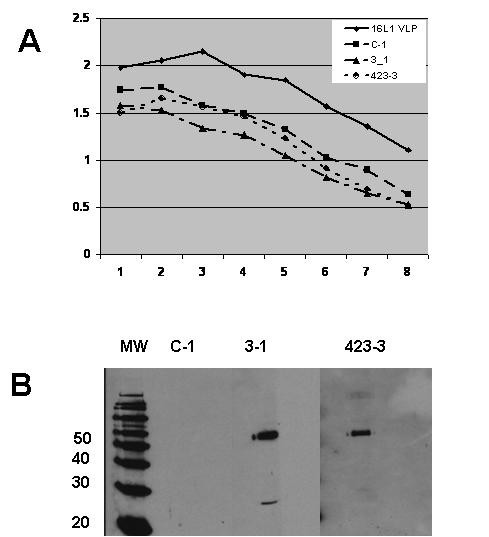
**Immunogenicity of L1 capsomere derivatives**. A) ELISA of L1 VLPs using antisera from mice immunized with L1 VLPs, or the capsomere derivatives 3-1, C-1 and 423-3. Horizontal axis represents serial two-fold dilutions starting at 1:5,000 and vertical axis represents OD_405 nm _of the resulting reactions. B) Immunoblot of purified RSV F protein using antisera from mice immunized with 3-1, C-1, or 423-3 (labelled on top of corresponding immunoblot lanes). Equal amounts of RSV F protein (approximately 0.5 μg/lane) were resolved on 10%/5% SDS-PAGE gels, transferred onto nitrocellulose, and detected with various mouse antisera (1:1,000 dilution) followed by goat anti-mouse IgG-HRP conjugate (1:10,000 dilution) and chemiluminescence. The sizes of the molecular weight standards are shown to the left of each marker ladder.

To determine whether C-1 capsomeres as well as the two derivatives bearing RSV F epitopes are immunogenic, we injected BALB/c mice with each of the three capsomere preparations emulsified with Freund's complete adjuvant (priming administration) and with Freund's incomplete adjuvant three weeks later (boosting administration); control mice were immunized on the same schedule with unadjuvanted L1 VLPs. Consistent with previous observations, week 6 sera from these mice exhibited robust immune response against L1 VLPs in ELISAs (Figure 5) and immunoblots (data not shown) [7,29]. As compared to the VLP-injected mouse sera, the slightly reduced VLP reactivity of sera from capsomere-injected mice may represent reduced immunogenicity of capsomeres as compared to capsids as previously described [12]. However, there was no obvious correlation between the anti-L1 immunoreactivity and the presence or absence of RSV F epitope within the h4 domain. With respect to reactogenicity against RSV F protein, sera from mice injected with 3-1 capsomeres and those immunized with 423-3 capsomeres recognized purified RSV F protein, whereas sera from C-1 injected mice bore no detectable anti-RSV F activity (Figure 5). However, none of the immune sera from capsomere-immunized mice recognized purified RSV F protein in ELISAs or bore RSV neutralizing activity (data not shown).

## Discussion

Because papillomavirus L1 protein-based oligomers (capsomeres and VLPs) can elicit a broad array of immune responses, they have been studied as potential vaccine platforms (reviewed in [15]). Such efforts have primarily focused on placing heterologous epitopes on surface-displayed, genetically variable loops of L1 or at the carboxy-terminus of full-length or truncated L1. Since capsomeres are also immunogenic, we tested the hypothesis that the h4 domain of the L1 monomer, which projects laterally and outwards on L1 pentamers, can be used for antigenic display of foreign epitopes. We demonstrate that: 1) such antigen presentation is feasible and does not overtly affect the formation of capsomeres; 2) such capsomeres likely exists as monomeric capsomeres with some degree of aggregation noted; and 3) mice immunized with capsomeres bearing RSV F epitopes generate antisera that recognizes the purified F protein. Thus, we conclude that foreign epitopes embedded within the h4 domain can be immunogenic when presented in the context of capsomeres.

Our findings have implications for structure of capsomeres. Two bacterially derived internal deletions spanning h4 – residues 410–427 and 404–436 that maintain or delete the h4-flanking prolines, respectively – can form capsomeres in vitro [6]. Based on these results, we initially produced similar deletions for our experiments. Unexpectedly, we noted that multiple efforts to purify B-1 (lacking 413–430 and bearing h4 flanking prolines) and its derivatives bearing the two RSV F epitopes consistently yielded capsomere preparations of limited quality and quantity. There are several possible explanations for this observation, including: subtle differences in the sequences deleted in our experiments as compared to those used by others; the use of baculovirus-derived L1 instead of prokaryotically expressed proteins; and structural constraints induced by the juxtaposition of several proline residues following internal h4 deletion.

In contrast, the deletion of the entire h4 domain and the surrounding proline residues (C-1, aa 404–436) led to purification and enrichment of capsomere derivatives, which were used in subsequent experiments. Furthermore, this L1 derivative formed capsomeres in the presence of two different heterologous epitopes of varying length and predicted structures (RSV F 255–278: helix-coil-helix 24 aa vs. RSV F 423–436: linear 14 aa). Since h4 projects laterally and outwards from capsomeres, its location may be more permissive to aa insertions as compared to other regions of L1. However, we did note a limited degree of capsomere aggregation in EM and sucrose gradient analyses. Such inter-capsomeric interactions may be dependent on the size and the primary and secondary structures of the foreign epitope within the h4 domain. Aggregation of capsomeres, if any, did not appear to affect the immunogenicity with respect to L1 epitopes.

Immunization of mice with capsomeres bearing RSV F epitopes elicited anti-F antibodies as tested by immunoblots using purified F protein. However, these antisera did not recognize purified RSV F on ELISAs and were non-neutralizing (data not shown). Since bacterially derived RSV F 255–278 fused to the carboxy-terminus of cholera holotoxin was immunogenic and protective, it is possible that the embedded presentation of the epitopes within the h4 domain may have altered the anti-F antibody response. We also cannot exclude the possibility that capsomere aggregation influenced the anti-RSV F response or that the immune response against RSV F sequences may have been subdominant to those on L1 surface loops. Lastly, the antigen processing and presentation may have been affected by the use of Freund's adjuvants.

## Conclusion

Our results serve as "proof-of-principle" studies to demonstrate that the h4 loop region of L1 can function as an antigen display site in the context of L1 capsomeres. Efforts to generate capsomere derivatives in prokaryotic systems, refine and improve the immunogenicity of modified L1 oligomers, and to expand the array of antigens for display, are in progress.

## Methods

### DNA constructions and manipulations

Plasmids and baculovirus stocks bearing HPV 16L1 cDNA have previously been described. Deletions of 16L1 cDNA were created by first ligating the entire cDNA of 16L1 into the BglII-SmaI site of pSP72 (Promega) to generate pSS1. Using PCR SuperMix High Fidelity (Invitrogen) and pSS1, inverse PCR was used to generate 16L1 derivatives deleted for aa 404–436 (del1) and aa 413–430 (del2). To enable modular oligonucleotide-based constructions, a unique NheI site encoding the residues ...AS... was engineered in place of each of the deletions. The following oligonucleotide 5' and 3' pairs (with the NheI site underlined) were used in PCR amplifications: del1: 5'GGCC**GCTAGC**AAAGAAGATCCCCTTAAAAAATATACTT; and 5'GGCC**GCTAGC**ATTCCAGTCCTCCAAAATAGT; del2: 5'GGCC**GCTAGC**CATACACCTCCAGCACCTAAAGAAGATC; and 5'GGCC**GCTAGC**GCCTCCTGGAGGAGGTTGTAAAC. The following PCR conditions were used: 94°C × 5 minutes × 1, then 35 cycles of 94°C × 1 minute, 55°C × 30 seconds, and 68°C × 5 minutes, followed by 68°C × 7 minutes and overnight storage at 4°C. The PCR amplicons were column purified (Qiagen), serially digested with NheI and DpnI (to remove the pSS1 template) and then self-ligated. The resulting plasmids were sequenced to confirm the existence of the respective deletions and NheI sites within the L1 cDNA. Thereafter, the modified L1 cDNAs were excised and ligated into the BglII-SmaI sites of pVL1392 (Orbigen), during which the NheI site remained unique within the resulting plasmids, termed pC-1 (bearing 16L1 del1) and pB-1 (bearing 16L1 del2).

To embed RSV F protein epitopes within the h4 domain of L1 derivatives, codon-optimized complementary oligonucleotides that encode aa 255–278 (SELLSLINDMPITNDQKKLMSNNV) and 423–438 (TASNKNRGIIKTFS) of RSV A2 strain F protein (GI: 333933) were used. These oligos bore NheI-compatible termini and were phosphorylated with T4 kinase (NEB), annealed, and ligated into the NheI site of pC-1 and pB-1. Sequencing of the resulting plasmids confirmed the existence of the appropriate oligonucleotide sequences and predicted to encode the aa sequence: AS...RSV F epitope...AS, in which the alanine and serine flanking the RSV-derived residues are derived from the NheI site. Note that the oligos were designed such that the first residue (S) of RSV F 255–278 starts from the serine incorporated from the NheI site, i.e. the amino terminus of the epitope-bearing sequence is: ...ASELL...

To construct the baculovirus stocks for expression of L1 proteins used in this study, pC-1 and pB-1 derivatives were co-transfected into *Spodoptera frugiperda *(*Sf9*; Invitrogen) cells with linearized baculovirus DNA (Baculo-Gold; BD Biosciences) and cellfectin (Invitrogen). After 72 hrs, the Sf9 serum-free media from each co-transfection was removed and the baculovirus stocks were serially propagated and plaque purified × 3 prior to use in *T. ni *cells.

### Protein expression and purification

RSV F protein was purified as previously described. Infection of *T. ni *cells with baculovirus bearing the 16L1 cDNA and subsequent purification of L1 VLPs were performed as described. The purification of capsomere derivatives was based on previous protocols and is briefly described as follows. *T.ni *cells growing at log phase in 250 mL cultures (1 – 2 × 10^6 ^cells/mL) in serum free media (Express Five, Invitrogen) were infected with each of the appropriate baculovirus stocks at a multiplicity of infection (MOI) of ≥ 3. After 72 hrs, the cells were collected by centrifugation, resuspended in ice-cold PBS + Complete Protease Inhibitor cocktail (Roche), and lysed using a Dounce homogenizer × 20 strokes and and a sonicator (3 × 20–30 second bursts, continuous cycle, output 3–4). The resulting mixture was brought to 40% CsCl (Roche) in 1× PBS and subjected to isopycnic ultracentrifugation at 28,000 × rpm × 40 hrs at 4°C using a Beckman SW28.1Ti rotor. The visible L1 band within the CsCl gradient was removed, dialyzed against PBS/0.5 M NaCl for >1 hr, and then overlayed onto a 30%/63% sucrose cushion using PBS/0.5 M NaCl as the solvent. After centrifugation at 28,000 × rpm × 5 hr at 4°C in a SW28.1Ti rotor, the capsomere-enriched fraction at the 0%/30% sucrose interface was removed and dialyzed exhaustively against PBS/1 M NaCl prior to -80°C storage and subsequent analysis.

### Immunological and structural characterizations of capsomere derivatives

For protein gel electrophoresis, capsomeres and L1 VLPs were mixed 1:1 with 2× SDS-sample buffer containing β-mercaptoethanol, heated at 95°C for 2–5 minutes, and then resolved on 10%/5% discontinuous SDS-PAGE using BioRad Protean Tetra-cell apparatus. Where appropriate, molecular weight markers (Novex and MagicMark; Invitrogen) were resolved in parallel and proteins were visualized by staining with Coomassie Brilliant Blue R-250. For immunoblots, the proteins were resolved on SDS-PAGE as above and then transferred onto nitrocellulose using a BioRad Trans-blot device (typically at 100V at room temperature for 1 hr). PBS + 0.1% Tween-20 (PBST) + 2% dried non-fat milk was used to block non-specific protein binding onto nitrocellulose. For detection of L1 protein, CAMVIR-1 (Santa Cruz Biotechnology) was used at 1:60,000 dilution followed by goat anti-mouse IgG heavy/light chain antibody-horseradish peroxidase (HRP) conjugate (Southern Biotech) at 1:20,000 in PBST. Antibody-antigen complexes were visualized by chemiluminescence (ECL; Pierce) and radiography (Kodak).

ELISAs were performed essentially as previously described. Typically, each protein for analysis was diluted with PBS and plated at 100 ng/well onto 96 well ELISA plates (Nunc) and incubated overnight at 4°C. Following incubation with primary antibodies as described in figure legends, alkaline phosphatase-conjugated goat anti-mouse secondary antibodies (Southern Biotech) and phosphatase substrate tablets (Sigma-Aldrich) were then used to visualize antigen-antibody complexes. The resulting colorimetric reactions were read at OD_405 nm _using a 96-well ELISA plate reader (Molecular Devices).

For electron microscopy analysis, capsomere samples (typically at 0.5–1 mg/ml in PBS/1 M NaCl) were diluted 1:10 in ice-cold PBS/1 M NaCl and then adsorbed onto carbon-coated grid for approximately 1 – 3 minutes. Excess fluid was then blotted with filter paper and the grids were negatively stained with 2% uranyl acetate. Images from grids were obtained using a Hitachi 7100 transmission electron microscope at 80 kV and 60,000 × – 100,000 × magnification. As control images for capsomeres, intact 16L1 VLPs were dissociated into capsomeres using previously described incubation conditions with dithiothreitol.

Sucrose gradients with standards (bovine catalase and *E. coli *β-galactosidase, Sigma-Aldrich; bovine serum albumin, VWR) were performed as described except that PBS/1 M NaCl was used as the sucrose solvent. Approximately 50 – 100 μg of each of the three standards were mixed with 100 μg of each of the capsomeres or intact L1 VLPs and subjected to ultracentrifugation at 41,000 × rpm × 16 – 20 hours at 4°C using a SW41.1Ti rotor (Beckman). The resulting gradient fractions (0.5 ml aliquots) were serially collected from the top of the ultracentrifuge tube, resolved on 10/5% SDS-PAGE, and then stained with Coomassie Brilliant Blue R-250 (for localization of standard peaks) or transferred to nitrocellulose and probed with CAMVIR-1 anti-L1 mAb prior to chemiluminescence detection.

### Immunogenicity of capsomere derivatives in mice

The animals were fed standard diet and water ad libitum and housed a pathogen-free environment within the University of Rochester School of Medicine and Dentistry Vivarium. Prior to any immunogenicity studies, all animal care and use protocols used in this study were approved by the Institutional Animal Care and Use Committee at the University of Rochester Medical Center. Female 6 – 8 week old BALB/c mice (Jackson Laboratories) in groups of 4 – 5 mice per capsomere were injected intramuscularly with 50 ug of each of the capsomeres. For priming injection, the capsomeres were diluted 1:1 and emulsified with Freund's complete adjuvant, while for boosts at weeks 3 and 6, Freund's incomplete adjuvant was used at 1:1 dilution. At week 6, submandibular bleeds were performed on the mice and the resulting sera were analyzed for reactogenicity against purified RSV F protein and purifed 16L1 VLPs. For antisera against 16L1 VLPs, 50 μg of purified VLPs were injected intramuscularly into mice as above except that no adjuvants were used.

## Competing interests

YM, RCR, and EEW are authors of a provisional patent application on the use of human papillomavirus L1 protein and its derivatives, including capsomeres, as RSV vaccine candidates.

## Authors' contributions

YM designed the experiments and drafted the manuscript. YM and PML performed all experiments except for purification of RSV F protein and RSV neutralization assays that were performed by EEW. RCR provided DNA for constructions and offered advice on capsomere purification and characterization. All authors read and approved the final manuscript.
